# RAB7 counteracts PI3K-driven macropinocytosis activated at early stages of melanoma development

**DOI:** 10.18632/oncotarget.4055

**Published:** 2015-05-09

**Authors:** Direna Alonso-Curbelo, Lisa Osterloh, Estela Cañón, Tonantzin G. Calvo, Raúl Martínez-Herranz, Panagiotis Karras, Sonia Martínez, Erica Riveiro-Falkenbach, Pablo-Ortiz Romero, José Luis Rodríguez-Peralto, Joaquín Pastor, María S. Soengas

**Affiliations:** ^1^ Melanoma Laboratory, Molecular Oncology Programme, Centro Nacional de Investigaciones Oncológicas (CNIO), Madrid, Spain; ^2^ Experimental Therapeutics Programme, Centro Nacional de Investigaciones Oncológicas (CNIO), Madrid, Spain; ^3^ Instituto de Investigación, Hospital 12 de Octubre Medical School, Universidad Complutense, Madrid, Spain; ^4^ Memorial Sloan Kettering Cancer Center, New York, USA

**Keywords:** macropinocytosis, endosomes, small GTPases, oncogene-induced senescence, oncogenic stress

## Abstract

Derailed endolysosomal trafficking is emerging as a widespread feature of aggressive neoplasms. However, the oncogenic signals that alter membrane homeostasis and their specific contribution to cancer progression remain unclear. Understanding the upstream drivers and downstream regulators of aberrant vesicular trafficking is distinctly important in melanoma. This disease is notorious for its inter- and intra-tumoral heterogeneity. Nevertheless, melanomas uniformly overexpress a cluster of endolysosomal genes, being particularly addicted to the membrane traffic regulator RAB7. Still, the underlying mechanisms and temporal determinants of this dependency have yet to be defined. Here we addressed these questions by combining electron microscopy, real time imaging and mechanistic analyses of vesicular trafficking in normal and malignant human melanocytic cells. This strategy revealed Class I PI3K as the key trigger of a hyperactive influx of macropinosomes that melanoma cells counteract via RAB7-mediated lysosomal degradation. In addition, gain- and loss-of-function *in vitro* studies followed by histopathological validation in clinical biopsies and genetically-engineered mouse models, traced back the requirement of RAB7 to the suppression of premature cellular senescence traits elicited in melanocytes by PI3K-inducing oncogenes. Together, these results provide new insight into the regulators and modes of action of RAB7, broadening the impact of endosomal fitness on melanoma development.

## INTRODUCTION

Deregulation of endocytosis, namely, vesicle generation from the plasma membrane, is raising considerable attention in the cancer field for its ability to modulate a variety of pro-tumorigenic signalling cascades [1, 2]. Endosomal uptake (particularly in large vacuolar structures or macropinosomes) can be exploited by neoplastic cells to scavenge lipids and proteins from the extracellular space and thus fuel biosynthetic pathways [3, 4]. In addition, multiple points of crosstalk have been described among endosomal factors and cell signalling and cytoskeleton regulators during tumor cell division, motility, invasion and metastatic dissemination [1, 5]. Intriguingly, aberrant vesiculation has also long been associated with suppressive programmes of premature senescence induced by various oncogenes, although the underlying mechanisms remain to be elucidated and may be cell type dependent [6-8]. In particular, the specific contribution of the endosomal machinery to early stages of tumor development is still unclear.

A main hurdle for the discovery of tumor drivers among endosome-associated factors is the very complexity of vesicle dynamics [2, 9]. Endovesicles can originate from the plasma membrane, the endoplasmic reticulum, the Golgi and/or from autophagosomal membranes [10, 11]. Moreover, endosomal cargo can be directed towards the lysosome for degradation, or sorted back to the plasma membrane [12]. Nevertheless, while endosomes and lysosomes are ubiquitously present in all tumor cells, we have recently reported an unexpected lineage-specific wiring of these organelles [13, 14]. Specifically, unbiased mining of transcriptomic databases revealed a cluster of endolysosomal genes that is selectively and uniformly enriched in melanoma cells in a manner not shared with over 35 different cancer types [13]. This unique co-regulation of endolysosomal genes was rather surprising considering that melanomas are a prototype of histopathologically heterogeneous tumors [15], where even the most frequent genetic alterations (i.e. oncogenic mutations in *BRAF*) show a varied penetrance [16-19]. However, expression and functional analyses of melanoma-enriched endolyososomal factors revealed a particular dependency of this tumor type on the membrane traffic regulator RAB7A.

RAB7A (herein referred to as RAB7 for simplicity) is a prototype of small GTPases that orchestrate vesicle trafficking by coordinating the fusion of endosomes and autophagosomes to lysosomes [20-22]. However, there is no consensus from the literature regarding the specific contribution of RAB7 to tumor progression. Thus, both pro-oncogenic [23, 24] and suppressive functions [25-27] of RAB7 have been reported in different cultured cell types, suggesting a highly context dependent action of the endolysosomal pathway in cancer. Moreover, mechanistic studies of RAB7 in human tumors are scarce, mostly limited to hormone secretion in thyroid cancer [28], or to as yet undefined functions in diffuse peritoneal malignant mesothelioma [29]. Intriguingly, we recently found that melanoma cell lines and tumors distinctly express high levels of RAB7 [13]. Furthermore, depletion of RAB7 significantly impacted the proliferation and morphology of melanoma cells*,* with minimal or qualitatively different effects observed in other cell types [13]. Promoter-based analyses and further validation in tissue specimens revealed the neural crest lineage master regulator SOX10 and the oncogene MYC as new upstream drivers of *RAB7* transcription in melanomas [13]. However, while SOX10 and MYC control a wide variety of cellular processes in melanoma cells [30-34], they have not been directly linked to endolysosomal trafficking. Therefore, alternative signaling cascades are likely to act upstream of RAB7 in melanoma cells. The identity of such signaling cascades and to which extend these pathways trace back to stress response mechanisms activated during melanoma development remain to be defined.

Here we performed a comprehensive characterization of vesicular trafficking in normal melanocytes and melanoma cells, clinical biopsies and mouse models to define when and how this tumor type becomes “addicted” to RAB7. This analysis revealed oncogenic Class I PI3K signaling as the upstream trigger of a hyperactive influx of plasma membrane-derived macropinosomes in melanoma cells that required RAB7 to be efficiently counteracted. This constitutive macropinocytic activity was retraced to primary melanocytes where PI3K-deregulating oncogenes were found to disrupt vesicular trafficking and elicit premature cellular senescence in a manner sensitive to the levels and functional status of RAB7. Together, our data identified a novel homeostatic role of RAB7 opposing oncogenic stress at early stages of melanocyte transformation, highlighting the relevance of the endolysosomal machinery on melanoma initiation and progression.

## RESULTS

### Selective modulation of RAB7-dependent vesicular trafficking in melanoma cells by pharmacological blockers of stress-response programmes

**Figure 1:**
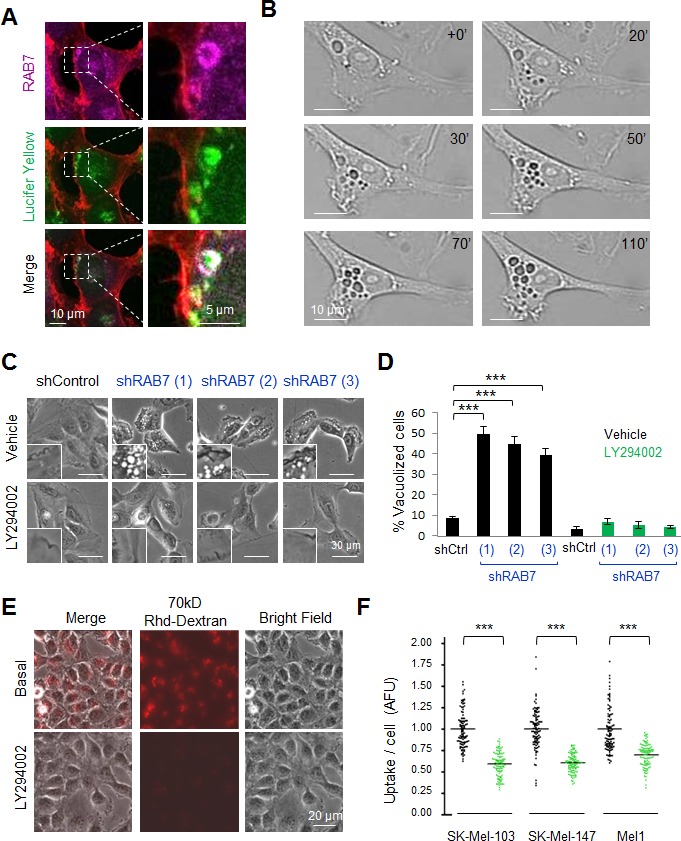
The pan-PI3K inhibitor LY294002 inhibits constitutive RAB7-regulated endosomal trafficking in melanoma cells **A.** Confocal fluorescence images of the incorporation of Lucifer Yellow (green) into RAB7-coated enlarged vesicles (pink) in SK-Mel-103 melanoma cells. Endogenous RAB7 protein was visualized by immunofluorescence (IF) staining. Phalloidin (red) staining outlines cytoskeletal actin. **B.** Snapshots of live imaging of SK-Mel-103 melanoma cells constitutively expressing RAB7 shRNAs, showing an active formation of large vacuolar structures ( > 1 μm diameter) from ruffling regions of the plasma membrane that accumulate in the perinuclear area. **C.** Bright field micrographs showing the reversion of shRAB7-driven cytosolic vacuolization by the pan-PI3K inhibitor LY294002 (10 μM, 8 h) in SK-Mel-103 melanoma cells transduced with three different RAB7 shRNAs. **D.** Quantification of the inhibitory effect of 10μM LY294002 on RAB7 shRNA-driven vacuolization of SK-Mel-103 assessed 24 h after treatment. Pooled data show means ± SEM of two independent experiments performed in duplicate. **E.** Bright field, fluorescence and merged micrographs showing the uptake of 70 kD Rhodamine(Rhd)-Dextran (8h) by SK-Mel-103 melanoma cells incubated in the absence or presence of 10μM LY294002. **F.** Confocal-based quantification of Lucifer Yellow uptake per cell (30 minute time frame acquisition), estimated in a minimum of 100 cells and expressed as arbitrary fluorescence units, AFU, with respect to non-treated cells.

We have previously reported that melanoma cells are particularly dependent on RAB7 to prevent the accumulation of large intracellular vesicles and the induction of an otherwise silent premature senescence program [13, 14]. In contrast, these traits were not observed in RAB7-depleted normal melanocytes [13]. Therefore, we hypothesized that the requirement for RAB7 may stem from oncogenic signals that deregulate vesicular trafficking to potentially harmful levels. To assess this hypothesis, we selected SK-Mel-103 as a representative example of aggressive melanoma cell lines with an endogenously active RAB7-dependent endocytosis (see Figure S1A and Figure 1A for visualization of the uptake of the fluid phase tracer Lucifer Yellow, and its incorporation into RAB7-decorated endosomes, respectively). In fact, blocking RAB7 function with validated short hairpin interfering RNAs (shRNAs) or the dominant negative mutant RAB7^T22N^ [13] prompts a dynamic accumulation of plasma membrane-derived macropinosomes in these cells (see Figure 1B, and Supplemental Video S1 for additional detail by time-lapse microscopy). This vesicle accumulation induced by RAB7 depletion was accompanied by a marked induction of classical (but yet incompletely understood) senescence-associated phenotypic traits such as β-galactosidase activity (SA-β-Gal) (Figures S1B,1C), as previously described [13]. A pharmacological analysis was then performed to identify oncogenic drivers of the vesicular trafficking that might be acting upstream of RAB7 and requiring this GTPase for their control. To this end, SK-Mel-103 cell were transduced with three independent shRNAs against RAB7 (to ensure robustness in the screening analysis). Once intracellular vacuolization was observed (see Materials and Methods), cells were treated with blockers of stress-inducing oncogenic pathways that are frequently deregulated in melanoma [19]. Particular attention was dedicated to inhibitors of MEK (U0126), PI3K (LY294002), Sonic Hedgehog (cyclopamine) or p38MAPK (SB20190). As vesicular trafficking can be also regulated by lysosomal and ubiquitin-dependent degradative pathways, additional analyses included modulators of protein degradation by the proteasome or the autolysosome (i.e. bortezomib and rapamycin, respectively) [35, 36]. Impact on RAB7-dependent macropinocytosis was defined by optical microscopy and by imaging the internalization of a classical macropinocytosis tracer (70kD-Dextran) labelled with Rhodamine for fluorescence-based detection [37]. Of the compounds tested, only the pan-PI3K inhibitor LY294002 was able to revert the aberrant vesicle accumulation driven by RAB7 depletion in an efficient manner within hours of treatment (Figure 1C, 1D; Figure S1D, and results not shown). In fact, consistent with the previously described roles of PI3K in membrane trafficking [38], LY294002 significantly inhibited the uptake of 70kD-Rhodamine-Dextran (Figure 1E) and Lucifer Yellow (see quantification in Figure 1F), further supporting inhibition of macropinocytosis. These results suggest a key role of PI3K in driving a hyperactivated macroendosomal influx in melanoma cells that is intrinsically opposed by the action of RAB7.

### Constitutive RAB7-dependent macropinocytosis of melanoma cells is triggered by Class I PI3K

**Figure 2:**
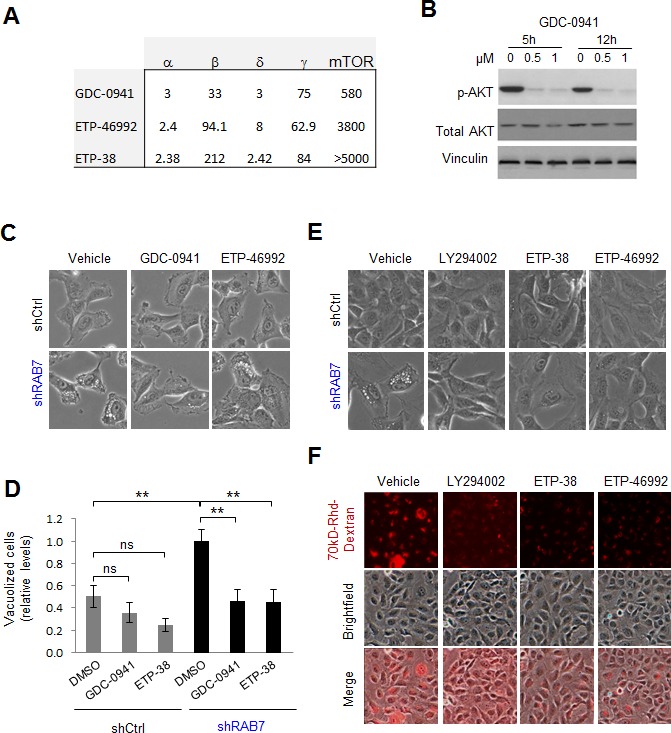
Class I PI3K signaling drives constitutive RAB7-regulated macropinocytosis in melanoma cells **A.** *K*_i,app_ values (in nM) of the indicated Class I PI3K inhibitors (see Methods for additional detail). **B.** Western blot analysis of SK-Mel-103 melanoma cells treated for the indicated times with Class I PI3K inhibitor GDC-0941 at the indicated concentrations, blotted for total and phosphorylated (p) AKT (Ser473). Vinculin is included as loading control. **C.** Representative bright field micrographs of shControl or shRAB7 SK-Mel-103 cells treated with DMSO (left) 0.5μM GDC-0941 (middle) or 0.5μM ETP-46992 (right) for 7h. The corresponding quantification of the impact of these treatments on cytosolic vacuolization is shown in **D. E.** Bright field micrographs showing the reversion of cytosolic vacuolization of SK-Mel-103 expressing RAB7 shRNAs by treatment with the indicated Class I PI3K inhibitors for 48h. **F.** Bright field, fluorescence and merged micrographs of the basal 8h-uptake of 70 kD Rhodamine(Rhd)-Dextran by SK-Mel-103 melanoma cells incubated in the absence or presence of the indicated Class I PI3K inhibitors for 48h.

Although LY294002 has been broadly used as a Class I PI3K inhibitor, this compound can also target other signalling cascades such as Class III PI3K [39]. Therefore, further analyses were performed with additional inhibitors targeting Class I PI3K more specifically. Given the complexity of Class I PI3K, with a catalytic p110 subunit constituted by one of four possible isoforms (α, β, δ and γ), in a heterodimeric complex with a regulatory subunit with also multiple variants [38], we opted for GDC-0941 [39], a well-known pan-p110 inhibitor (see pharmacological features of this compound in Figure 2A). Dose-response and kinetic analyses were performed in SK-Mel-103 to identify minimal effective concentrations for an efficient blockade of PI3K signalling, as defined by the abrogation of AKT phosphorylation in residue Ser473 (Figure 2B). As shown in Figure 2C, GDC-0941 reverted very efficiently the vacuolization induced in melanoma cells by RAB7 depletion (see the corresponding quantifications in Figure 2D). To independently validate these results, RAB7 shRNA-expressing melanoma cells were treated with ETP-46992 [40], a structurally different Class I pan PI3K blocker with an even more selective inhibitor profile (i.e. with reduced affinity for other kinases such as mTOR; see Figure 2A). ETP-46992 also resolved the aberrant vacuolization induced by RAB7 shRNA (Figure 2C, 2D). Importantly, ETP-38, a derivative of ETP-46992 (Figure 2A) that selectively binds and inactivates p110α and p110δ [41], also effectively rescued shRAB7-induced vacuolization (Figures 2E, 2D) and impaired constitutive macropinocytosis (Figure 2F), further narrowing down the p110 subunits that deregulate vesicular trafficking in a manner that is sensitive to RAB7 inhibition. Therefore, these data point to p110α and p110δ Class I PI3K as key activators of RAB7-dependent macroendocytic trafficking in melanoma cells.

### Oncogene activation triggers RAB7-dependent macropinocytosis in primary human melanocytes

PI3K pathway activation is an early event in melanoma development [15]. This signalling pathway can be activated directly by deregulated RAS oncogenes (mutated in about 25% of melanomas), or indirectly, for example as a result of *PTEN* loss (the latter commonly found in *BRAF*-mutated melanomas, which constitute 50-60% of the cutaneous forms of this disease) [42]. Therefore, we next sought to address whether the dependency of melanoma cells on RAB7 for counteracting PI3K-driven vesicle trafficking was established early during tumor progression, i.e. at the level of oncogene activation in normal human melanocytes. To this end, fresh preparations of primary melanocytes were transduced with lentiviral vectors encoding for HRAS^G12V^, here used as a prototypical tool to assess PI3K-associated stress response programs [43] (results with activated NRAS and BRAF are presented below). In particular, we and others have previously shown that normal melanocytes are highly sensitive to HRAS^G12V^, responding with marked vacuolization and induction of lysosomal SA-β-Gal activity in a PI3K-dependent manner [8, 33]. Still, as in other cell types, the mechanism(s) underlying the induction of these classic phenotypic traits in the context of vesicular trafficking remain unclear. Moreover, HRAS^G12V^ is relevant in the cutaneous oncology field as it mimics alterations found in Spitz nevi, a histopathologically heterogeneous melanocytic lesion that may be challenging to diagnose [44].

**Figure 3:**
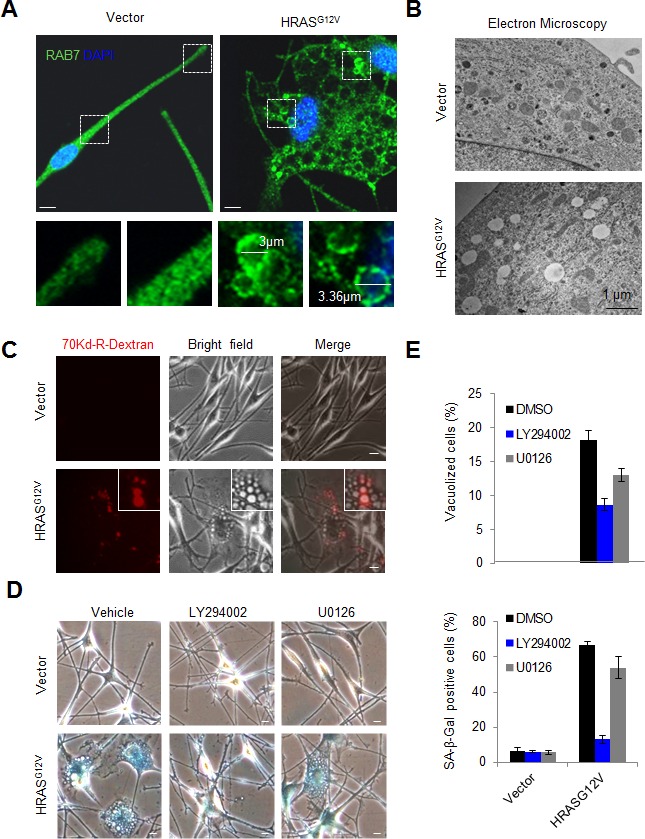
Recruitment of RAB7 to PI3K-driven macropinosomes upon oncogene activation in human melanocytes **A.** Representative IF staining for RAB7 (green) in primary human melanocytes expressing empty vector or oncogenic HRAS^G12V^. Nuclei are counterstained with DAPI. **B.** Transmission electron microscopy image of primary human melanocytes expressing empty vector or HRAS^G12V^. Note the presence of large single membrane macropinosomes in HRAS^G12V^-expressing melanocytes, absent in the empty vector-expressing counterparts. **C.** Representative fluorescence, bright field and merged micrographs showing the activation of macropinocytic uptake (visualized by 70kD Rhodamine(Rhd)-Dextran uptake) in primary human melanocytes expressing oncogenic HRAS^G12V^. **D.** Representative bright field micrographs showing senescence-associated β-Galactosidase (β-Gal) stainings of melanocytes transduced with empty HRAS^G12V^-encoding vectors and treated with 10μM LY294002, 10 μM U0126 or vehicle control. Inhibitors were added one day post-transduction and were refreshed every 24h. Pictures were taken at day 6 post-transduction. **E.** Pooled quantification of vacuolized and β-Gal-positive cells from two independent experiments in vector- or HRAS^G12V^- expressing melanocytes treated as indicated. Unless otherwise indicated, scale bars correspond to 10 μm.

Immunofluorescence analyses of endogenous RAB proteins and endolysosomal markers (i.e. LAMP1) revealed an overt deregulation of the endolysosomal pathway in HRAS^G12V^-expressing melanocytes, characterized by recruitment of RAB7 to the enlarged vacuolar structures induced by this oncogene (Figures 3A, S2A and results not shown). These large RAB7-positive vacuoles could originate from the plasma membrane, or from intracellular organelles such as the endoplasmic reticulum, the Golgi, and/or various recycling endomembranes [10, 45, 46]. In addition, RAB7 controls the fate of double-membrane autophagosomes [21], of relevance in oncogene-induced senescence in embryonic fibroblasts [47]. Addressing these different sources is important as a variety of key pathways involving and cooperating with RAS-dependent effectors can signal in a differential manner depending on their membrane-associated localization [48]. To this end, HRAS^G12V^ transduced melanocytes were processed for imaging by electron microscopy for a direct analysis of the ultrastructural cellular changes driven by this oncogene. This technique revealed that over 80% of vacuoles in HRAS^G12V^-transduced melanocytes in fact corresponded to single-membrane vesicles (Figure 3B). The size of these vesicles, from 0.2 to over 2 μm (Figures 3A, 3B), their ability to uptake large solutes (70kDa-Dextran) from the extracellular space (Figure 3C), as well as their formation from actin-rich membrane ruffling (Figure S2B), support plasma-membrane driven macropinocytosis as the source of oncogene-driven vesicles that recruit RAB7 downstream of HRAS^G12V^. As both these oncogenic signals and macropinocytosis are absent in normal melanocytes, these results provide further mechanistic evidence as to why melanomas are significantly more dependent on RAB7 than their normal cellular counterparts [13].

### RAB7 modulates Oncogene-Induced Senescence (OIS) in melanocytes downstream of PI3K-activating oncogenes

Consistent with an oncogene-induced senescence program [7], HRAS^G12V^-expressing melanocytes not only accumulated large RAB7-positive macroendocytic vesicles but also became characteristically blue when stained for SA-β-Gal (Figure 3D). We then determined whether these RAB7-associated “vesicular traits” of senescent melanocytes were dependent on active PI3K signalling (i.e. instead of the BRAF > MEK > ERK pathway as described for HRAS^G12V^-driven OIS in other primary cell types [49, 50]). To this end, melanocytes were treated with LY294002 or the MEK inhibitor U0126, starting 24h after lentiviral-driven transduction of HRAS^G12V^ (preceding cell cycle arrest). While U0126 could reduce cellular vacuolization, this effect was more potent for LY294002, which also showed a significantly stronger inhibitory effect on SA-β-Gal staining (see micrographs in Figure 3D and quantification in Figure 3E). Therefore, these results link PI3K-pathway activation to the massively hyperactivated RAB7-associated macropinocytosis observed in HRAS-transduced melanocytes.

**Figure 4:**
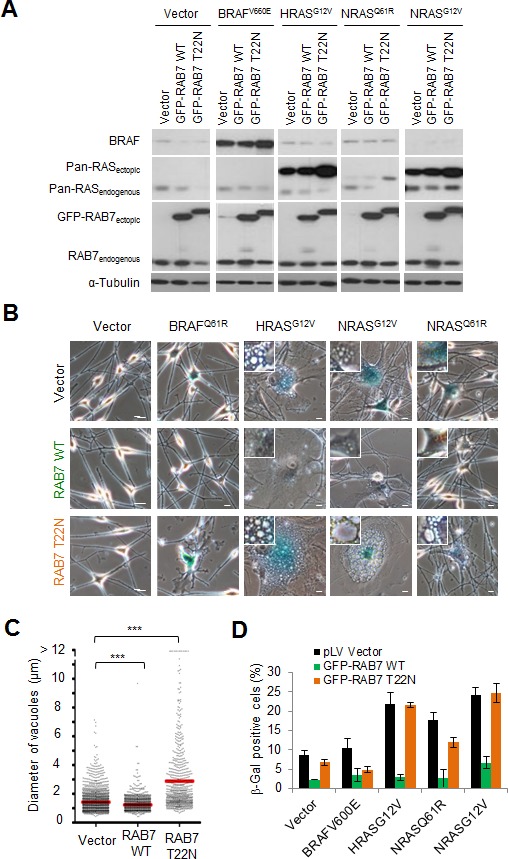
RAB7 counteracts PI3K-driven oncogenic stress **A.** Immunoblot analyses of total cell extracts isolated from melanocytes co-expressing the indicated oncogenes and wild-type (WT) or dominant negative (T22N) GFP-RAB7, or their corresponding empty vector controls. **B.** Representative micrographs showing the effect of RAB7 wild-type (WT) or dominant negative (T22N) overexpression on SA-β-gal staining and cytoplasmic vacuolization in primary human melanocytes expressing the indicated oncogenes. Scale bars, 10μm. **C.** Dot plot showing the impact of RAB7 wild-type (WT) or dominant negative (T22N) overexpression in the size of HRAS^G12V^-induced vacuoles in primary human melanocytes (vacuoles of ≥1μm in diameter were individually measured). **D.** Quantification of SA-β-gal positive cells from **B.**. Data are presented as means ± SEM of three independent experiments.

**Figure 5:**
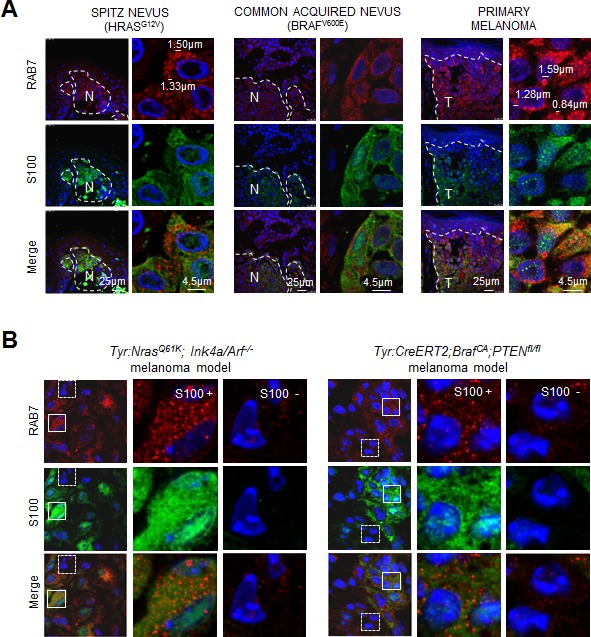
*In vivo* visualization of RAB7-positive macropinosomes **A.** Co-staining of RAB7 (red) and S100 (green) in paraffin-embedded sections of the indicated human melanocytic skin lesions. **B.** Visualization of RAB7-decorated endosomes (red) in paraffin-embedded melanocytic lesions (marked in green by S100) generated in indicated mouse models. Note high vesicular-patterned staining of RAB7 in S100-positive (S100+) melanocytes compared to S100- skin cells.

Next we questioned whether the deregulation of RAB7-controlled pathways downstream of PI3K activation was just a passive inconsequential byproduct of stress-inducing pathways or, in contrast, had an active role in modulating OIS. To this end, RAB7 function was induced or repressed in oncogene-expressing melanocytes by ectopic expression of wild type RAB7 or the dominant negative RAB7^T22N^ mutant, respectively. Both RAB7 constructs were fused to GFP for real time fluorescence imaging. Importantly, these analyses were performed in melanocytes expressing HRAS^G12V^, as well as oncogenic forms of NRAS (i.e. NRAS^G12V^ or NRAS^Q61R^), the latter being characteristic of congenital nevi and a sizable fraction (about 25%) of melanomas [15]. BRAF^V600E^ was also analyzed in parallel as an OIS inducer that is mechanistically different to H/NRAS, with no activation of PI3K signalling nor induction of obvious vesicle-associated phenotypes in normal melanocytes [8]. The relative expression of the GFP-RAB7^T22N^ and the different oncogenes is summarized in Figure 4A. As shown in Figure 4B, the functionally inactive RAB7^T22N^ exacerbated RAS-induced macropinocytosis, aberrant vacuole accumulation and positive SA-β-Gal activity (see also Figure S3A). Conversely, overexpression of ectopic wild type RAB7, which would mimic the induction of this protein found at early stages of melanoma initiation [13], was found sufficient to suppress RAS-driven OIS features. Specifically, wild type RAB7 favored the clearance of oncogene-driven macropinosomes, preventing the accumulation of large cytosolic vacuoles (Figures 4B, 4C, S3A), and significantly abrogated SA-β-Gal activity (Figure 4B, 4D). In contrast, BRAF^V600E^-driven OIS -not activating PI3K directly (Figure S3A), neither inducing macropinocytosis (Figure S3B), was not significantly affected by RAB7 overexpression or functional inactivation (Figures 4A-4D). Additional PI3K-activating events (i.e. PTEN loss) in BRAF^V600E^-expressing melanocytic lesions did indeed trigger RAB7-regulated macropinocytosis (see below in Figure 5). Together, these results support the concept of an active selection of RAB7 upregulation already at very early stages of melanocyte transformation to counteract an otherwise potentially damaging “endosomal surplus” driven by derailed PI3K signalling.

### RAB7-regulated vesicular trafficking visualized *in vivo* at early stages of melanoma development

A corollary of the results presented above in cultured cells, is that RAB7 protein should be mobilized to macropinosomes in H/NRAS-driven nevi, to be further induced at early-stage malignant lesions, where OIS is suppressed or bypassed to allow for melanoma development [42]. To validate this hypothesis *in vivo*, the levels and subcellular distribution of endogenous RAB7 were analyzed in Spitz nevi (representing senescent RAS-deregulated lesions), common acquired nevi (representing BRAF^V600E^-induced senescent lesions) and RGP primary cutaneous melanomas. Tissues were also co-stained for S100 to mark melanocytic cells within the lesions. These analyses revealed S100 positive cells of Spitz nevi containing large vesicular structures decorated by RAB7 (Figure 5A, top left panels), highly reminiscent of the RAB7-positive macropinosones observed in cultured oncogene-expressing senescent melanocytes (Figure 3A, 3C). Importantly, these features of RAB7-dependent macropinocytosis were absent in benign BRAF^V600E^ lesions (Figure 5A, middle panels), but were markedly upregulated in their malignant counterparts (Figure 5A, top right), likely reflecting frequent PI3K-activating events associated with melanoma development [42].

As human melanocytic lesions are genetically complex, we set to assess the levels and cytosolic distribution of RAB7 in genetically-engineered mouse melanoma models that allow for a more direct analysis of vesicular trafficking downstream of oncogenic PI3K *in vivo*. In particular, we used two well-characterized models harbouring constitutively activated PI3K signaling in melanocytes downstream (i) mutated Nras (*Tyr:Nras^Q61K^;Ink4a/Arf^−/−^*) [51] or (ii) Pten loss, the latter in a melanoma-prone background of constitutively active BRAF^V600E^ (*Tyr::CreERT2;BrafCA;Pten^fl/fl^*) [52]. As summarized in Figure 5B, early melanoma lesions developed in both mouse models showed RAB7 recruitment to enlarged vesicles, specifically in the oncogene-expressing melanocytic cells and not in the surrounding stroma (compare S100 positive and negative areas in Figure 5B). As RAB7 is anchored to membranes in its active GTP-bound form [20], these results provide physiological evidence of direct involvement of this GTPase in the turnover of oncogene-driven macropinosomes at early stages of melanoma development.

## DISCUSSION

Lysosomal-dependent degradation has long attracted attention in oncology for its putative impact on a variety of signalling cascades that control tumor cell proliferation, invasion and response to therapy [1, 48]. While there is abundant information about lysosomal functions in the context of double membrane autophagosomes [53], regulators and effectors of single membrane endosomes are still incompletely understood. This limited information is particularly relevant in the light of recent reports by our group and subsequently by others, revealing a particular hyperactivation of the endolysosomal machinery in melanoma cells [13, 54]. The small GTPase RAB7 in fact, shows the highest enrichment in melanoma, where it is essential to sustain cell proliferation [13]. Yet, the molecular determinants linking this requirement to aberrant vesicular trafficking are unknown. Here we used a combination of functional studies in normal- and oncogene-transduced melanocytes, as well as human melanoma cell lines, clinical biopsies and mouse models with a triple objective: (i) define the identity of membrane-trafficking deregulators acting upstream of RAB7, (ii) determine the stage during melanoma genesis when this dependency is established, and (iii) dissect the signalling cascades involved. These studies revealed aberrant PI3K signalling as the driver of an otherwise potentially damaging macroendosomal surplus that melanoma cells counteract via RAB7-mediated lysosomal degradation. This requirement of RAB7 was traced back to PI3K-driven oncogenic stress programmes activated at early stages of melanoma initiation. In particular, RAB7 function was found to oppose stress-associated features such as aberrant cytosolic vacuolization and lysosomal SA-β-Gal activity induced in primary human melanocytes downstream PI3K pathway activation, shedding light into the regulation of these classic hallmarks of oncogene-induced senescence (OIS). Furthermore, the mobilization of RAB7-regulated macropinosomes downstream of oncogene activation was demonstrated *in vivo*, further underlying RAB7 as a physiologically relevant homeostatic control of membrane dynamics in melanoma.

Narrowing down the drivers of RAB7-regulated vesicular trafficking to Class I PI3K is relevant considering that melanomas accumulate a plethora of (epi)genetic alterations. For example, BRAF > MEK, mTOR, p38, SHH and as well as proteasome-dependent proteolysis, are some of multiple pathways which can impinge on or can be influenced by vesicular trafficking [16-19]. Therefore it is intriguing that among inhibitors of these cascades, only Class I PI3K inhibitors were sufficient to revert the aberrant vacuolization of RAB7-depleted melanoma cells. Interestingly, from the different sources of endomembranes in mammalian cells (i.e. the plasma membrane, the ER, the Golgi or the autophagy machinery) [10, 45, 46], electron microscopy and real-time analyses of membrane trafficking revealed macropinocytosis as the main responsible of the aberrant endosomal surplus induced in melanoma cells upon RAB7 suppression. While PI3K controls vesicle generation and maturation in multiple systems [38], what was striking was not just the origin, but actually the extent of the macropinocytic influx found here by time lapse microscopy in melanoma cells. These results further highlight our previous findings of melanoma as a tumor type particularly “addicted” to endolysosomal degradation [13]. In this context, as PI3K activity is activated in a broad spectrum of cancer types, it would be interesting to identify late endosomal modulators (i.e. Cul3 [55]) that may compensate for low RAB7 levels in these non-melanoma cellular lineages.

Perhaps one of the most unexpected results of this study is that altering the levels and activity of a single membrane traffic regulator (RAB7) is sufficient to significantly alter the response of normal melanocytes to potent drivers of oncogene-induced senescence (OIS) such as NRAS or HRAS. Moreover, our data provide insight into two classical features of OIS, namely, aberrant cytosolic vacuolization and SA-β-Gal activity [6], which remain puzzlingly undefined in the cancer field. Thus, we show that the characteristic cytosolic vacuoles that are induced in senescent RAS-expressing primary melanocytes [8, 56] are, in fact, RAB7-positive macroendosomes. Moreover, it is tempting to speculate that the hyperactivation of lysosomal functions to counteract this endosomal surplus, may account for the increased lysosomal β-Galactosidase activity that marks primary senescent cells as characteristically blue at acidic pH [6]. Whether RAB7 (or functional analogues) mediate OIS-dependent lysosomal programs in other tumor types deserves attention.

Adding endolysosomal functions to OIS expands the contribution of membrane-associated events in the outcome of oncogene activation. We have previously reported that the H/NRAS oncogenes promote an acute Unfolded Protein Response (UPR) in melanocytes, with marked expansion of membranes of the endoplasmic reticulum [8], an essential organelle in secretory programmes [57]. In fact, a defining feature of OIS (in melanocytes and other cell types) is the secretion of a plethora of cytokines, matrix remodelers and other factors, termed as the senescence-associated secretory phenotype (SASP), which together blunt oncogenic transformation [58]. In addition, autophagy, has been also shown to modulate OIS, although the underlying mechanisms need to be fully elucidated and that may be context-specific [47]. This study now shows that, in addition to exocytic and autophagic processes, endocytosis should be considered when factoring active contributors to OIS. Defining the specific cargo of RAB7-regulated vesicles deserves further investigation, but it is tempting to speculate that, by regulating endosome fate, RAB7 function might directly impinge on the secretory phenotype of senescent melanocytic cells. Indeed, halted endolysosomal trafficking can be coupled with increased protein secretion in melanoma cells [13], and many critical SASP factors are known to be transported to the extracellular space from within the endosomal compartment [59]. Similarly, it would be interesting to explore putative cooperative interactions between RAB7 and frequently mutated melanomas drivers (e.g. RAC, cKIT, WNT), whose half-life and localization are also membrane trafficking-dependent [19].

This study also further emphasizes the differing roles of RAB7 in normal and melanocytic cells. While normal melanocytes can sustain RAB7 depletion without the acquisition of senescence-associated traits [13], here we show that is not the case once they acquire pro-tumorigenic mutations. In this context, the need for high RAB7 levels to counteract the enhanced PI3K-driven macropinocytic influx of oncogene-expressing melanocytic cells provides a plausible explanation for the selection of cooperating events (e.g. SOX10 and MYC induction) that increase the overexpression of this GTPase already at the stage of melanoma initiation [13]. Consistent with this scenario, inactivation of the RAB7 transactivator MYC in melanoma cells can re-activate dormant senescence programs reminiscent of OIS in melanocytes [33]. Events activating PI3K in mutated NRAS and BRAF-driven melanomas (i.e. PTEN loss) [42], further expand the requirement of RAB7 and the roles of macropinocytosis (e.g. scavenging nutrients such as albumin and amino acids, lipids and extracellular ATP [3, 4]) to a broader spectrum of melanocytic lesions.

From a clinical perspective, the visualization of large vesicular structures that recruit RAB7 *in vivo* (i.e. in human and murine nevi and melanomas) demonstrates that macropinocytosis is not just a cell culture artefact. Moreover, these data offer an attractive platform for therapeutic intervention. In particular, the differential ability of normal melanocytes and oncogene-bearing melanocytic cells to internalize large extracellular solutes already at early stages of tumor development provides an attractive platform for selective drug uptake in the context of cancer therapy. Thus, the literature is blooming with peptide-modified chemotherapeutic agents, lipid-based drug formulations and an increasing list of nanoparticles and polyplexes whose delivery largely depends on endocytosis (see Ref [60] for a review). Therefore, our data supports melanoma cells as an ideal scenario to test and validate these agents. As melanomas accumulate a myriad of (epi)genetic alterations, current treatments actively pursue combination therapies [61]. In this case, our data would argue that caution should be exerted when administering endosome-incorporated agents concomitant with Class I PI3K blockers (i.e. as this could reduce the effective intracellular drug concentration). In turn, compounds that exacerbate endo/lysosomal activity may be particularly effective in melanoma cells, as we have already reported for dsRNA-based nanoplexes [62].

In summary, in this study we demonstrate Class I PI3K-dependent hyperactive macropinocytic influx in melanoma cells as a source of endosomal vesicles that recruit and depend on RAB7 for their degradation. This RAB7-controlled vesicular trafficking was found to be hyperactivated at very early stages of melanoma development, uncovering a new homeostatic role of RAB7 counteracting an “endosomal surplus” associated with tumor suppressive oncogene-induced senescence. As macropinocytosis is actively pursued in pharmacological settings, our data offers a platform for therapeutic intervention not only in metastatic melanomas, but also to control incipient tumors before their dissemination to distal organs.

## MATERIALS AND METHODS

### Cells

Primary human melanocytes were isolated from neonatal foreskins (obtained from the Hospital Niño Jesús, Madrid, Spain), and cultured as described [8] in Medium 254 supplemented with melanocyte growth factors (HMG-1) containing 10 ng/ml phorbol 12-myristate 13-acetate (Invitrogen). SK-Mel-103 melanoma cells were cultured in Dulbecco's modified Eagle's medium (Invitrogen; Carlsbad, CA, USA) supplemented with 10 % fetal bovine serum (Lonza, Basel, Switzerland).

### Lentiviral-mediated gene transfer for RAB7 loss of function or gain of function

RAB7 function was stably inhibited by lentiviral mediated gene transfer using two approaches: (i) pLKO-based transduction of three previously validated shRNA (here in named as shRAB7 -1, 2 -and -3, targeting the sequences TAGGAGCTGACTTT, TTTCCTGAACCTAT, GATTGACCTCGAAA, respectively), purchased from Sigma (St Louis, MO, USA); and (ii) pLV-driven stable overexpression of the well-described RAB7 dominant negative mutant RAB7 (T22N) [63], fused to eGFP for visualization by fluorescence microscopy. RAB7 gain-of-function assays were performed by lentiviral overexpression of eGFP-RAB7 WT. pLKO expressing scrambled shRNA, and the empty pLV vector, vector were used as controls. All lentiviral infections were performed as previously described [8], validating gene transfer or knockdown by protein immunloboting, quantitative PCR or fluorescence imaging. Unless otherwise indicated, cells were plated for expression and functional assays after puromycin selection (1 μg/mL), at day 6 post-lentiviral infection.

### Protein immunoblotting

To determine relative differences in protein levels, 2×10^6^ cells were harvested at the indicated time points. Protein samples extracted from total cell lysates using Laemmli buffer were subjected to electrophoresis in polyacrylamide SDS gels under reducing conditions, and subsequently transferred to Immobilon-P membranes (Millipore, Bedford, MA, USA). Protein bands were detected using the ECL system (GE Healthcare, Buckighamshire, UK). Primary antibodies included: RAB7 (Clone RAB7-117); α-Tubulin (clone DM1A) and Vinculin (V9131) from Sigma (St Louis, MO, USA); pan-Ras (Pan-Ras (Ab-3)) from Calbiochem; AKT and p-AKT (Ser473) from Cell Signaling (Danvers, MA, USA); HRP-conjugated secondary antibodies were from GE Healthcare (Little Chalfont, Buckinghamshire, UK); and anti-goat-HRP, from Jackson Immunoresearch (West Grove, PA, USA). α-Tubulin or Vinculin blots were used as loading controls.

### Visualization and quantification of endocytosis in melanoma cells

Ultrastructural analyses of intracellular vesicles in melanoma cells were performed by transmission electron microscopy using a Philips CM-100 microscope, as previously described [62]. To visualize bulk fluid phase endocytosis by fluorescence microscopy, the indicated cellular populations were incubated in pre-warmed growth medium containing 1 mg/mL Lucifer Yellow (Sigma; St Louis, MO, USA) for 30 minutes. Alternatively, cells were incubated with 2 mg/mL 70000 Da Rhodamine-labeled dextran (Invitrogen, Carlsbad, CA, USA), for a more specific analysis of macropinocytosis [37]. After incubation with these markers, cells were washed and fixed with 4% paraformaldehyde. The incorporation of Lucifer yellow was visualized under a TCS-SP5-WLL (AOBS-UV) spectral microscope (Leica Microsystems, Wetzlar, Germany). Rhodamine-Dextran was visualized under a Nikon ECLIPSE TiE fluorescence microscope (Izasa, Barcelona, Spain). OPERA HCS platform and the Acapella Analysis Software were used for single-cell quantification of dextran uptake. For quantification of cytosolic vacuolization, cells were fixed with 4% PFA at the indicated time points, and a minimum of 200 cells per condition were scored according to the number and size of vacuoles. To assess the contribution of the PI3K pathway on RAB7-modulated macropinocytosis, melanoma cells were transduced with lentiviral vectors coding for control shRNA or RAB7 shRNA. 7 days after infection, cells were left untreated or were incubated with 10 μM LY294002 (Calbiochem), a classical PI3K inhibitor [39]. Alternatively, treatments were performed with the pan-p110 inhibitor ETP-46992 (CNIO), or with the p110α/δ blockers GDC-0941 (Axon Medchem) or ETP-38 (CNIO). Inhibitory constants against p110α, β, δ, γ and mTOR of these last three compounds are summarized in Figure 2A (see refs [39-41] for additional information on the structure and pharmacological profiles of these agents). Dosing of the PI3K inhibitors is indicated in the corresponding legends of Figure 2. All experiments were done in triplicate and were repeated at least twice. Pooled quantification data are presented as means ± SEM of two independent experiments. Treatments with 5 μM U0126, 25 nM Rapamycin, 5 μM SB20219 or 10 μM Cyclopamine to inhibit MEK, mTOR, p38 or SHH pathways, respectively, are depicted in Figure S1D. Time lapse videomicroscopy was performed using the Delta Vision RT microscope (Applied Precision, Washington, USA) coupled to a CO_2_ and temperature-controlled incubation chamber to allow for short- and long-term imaging of living cells.

### Oncogene-induced senescence assays (OIS) in HRAS^G12V^, BRAF^V600E^, NRAS^Q61R^ and NRAS^G12V^-expressing primary melanocytes

Primary human melanocytes were transduced with validated HRAS^G12V^, BRAF^V600E^, NRAS^Q61R^ and NRAS^G12V^-expressing vectors, as previously described [8]. The differential impact of PI3K vs MEK inhibition in OIS was performed by incubating HRAS^G12V^-transduced melanocytes with 10 μM LY294002 or 5 μM U0126, added 24 h after lentiviral infection following previously validated protocols [8]. To address the role of RAB7 in OIS, two sequential infections of 5h each were performed, first with GFP-RAB7 wild-type or T22N viral supernatants and secondly with oncogenic RAS- or BRAF–coding lentivirus. Melanocytes transduced with empty vectors were also included as wild type controls (i.e. not expressing oncogenes nor wild-type or dominant negative RAB7). Infection efficiencies were estimated at day 6 after infection by imaging of green fluorescence protein and by Western blot using the appropriate antibodies. To address macropinocytic trafficking, melanocytes were transduced with HRAS^G12V^-coding lentiviruses, and 6 days post-infection were incubated with 70 kD Rhodamine(Rhd)-Dextran (2 mg/mL) for 2.5 h. Cells were then washed, fixed with 4% paraformaldehyde and imaged under a Nikon ECLIPSE TiE fluorescence microscope or a TCS-SP5-WLL (AOBS-UV) spectral microscope. Actin-driven ruffling and endolysosomal trafficking were visualized by phalloidin and RAB7 and LAMP1 immunofluorescence staining, respectively, using a TCS-SP5-WLL (AOBS-UV) spectral confocal microscope. Cells were processed for immunofluorescence and SA-β-Gal staining at day 6 post-infection, as previously described [8]. Cytosolic vacuolization was quantified by scoring the number of vacuolized cells and the size of vacuoles (≥ 1 μm diameter) using a Nikon ECLIPSE TiE fluorescence microscope (Izasa, Barcelona, Spain) and the Nikon NIS-Elements BR software. Pooled quantification data of percentage of β-Galactosidase positive or vacuolized cells are presented as means ± SEM of two independent experiments.

### Immunofluorescence analyses of RAB7 *in vivo*

To visualize RAB7-decorated vesicles in human samples, melanoma and nevi biopsies were obtained from the i+12 Biobank (RD09/0076/00118) of the Hospital 12 Octubre and the Spanish Hospital Biobank Network, under appropriated ethical protocols by their Clinical Investigation Ethical Committees. Paraffin-embedded whole-tissue sections from Spitz nevi, common acquired nevi or malignant melanomas were histopathologically evaluated by two independent dermatopathologists. Tissue sections processed for immunofluorescence as previously described [13], using RAB7A (Prestige Antibody, powered by Atlas Antibodies) purchased from Sigma (St Louis, MO, USA); and S100 (Ab-1, Clone 4C4.9) from Thermo Scientific (Fremont, CA, USA) as primary antibodies. Nuclei were counterstained with DAPI (Invitrogen). The fluorescence emission was acquired using a confocal TCS-SP5-WLL (AOBS-UV) spectral microscope (Leica Mycrosystems, Wetzlar, Germany).

To visualize RAB7-decorated vesicles in murine melanoma samples, endogenous melanomas were generated in the melanocyte-specific *Tyr:CreERT2; Braf^V600E^/Pten^loxP/loxP^* and *Tyr:Nras^Q61K^;Ink4a/Arf^−/−^* mouse models as previously described [52, 62, 64]. Tumors were surgically excised when reaching a diameter of 1 cm, were processed for histology. Melanoma was confirmed by Trp1/Trp2 immunohistochemical staining and histological analysis by a pathologist. Immunofluorescence in murine tissue samples was performed as previously described [13] but using M.O.M Mouse IgG Blocking Reagent (purchased from Vector Laboratories; Burlingame, CA, USA) and Image-iT FX signal enhancer (from Invitrogen; Carlsbad, CA, USA) before the primary antibody incubation according to manufacturers' protocols. All animal experiments met the Animal Welfare guidelines and were performed in accordance with protocols approved by the Institutional Ethics Committee of the CNIO.

### Statistical analyses

The differences between two groups were evaluated by the two-tailed Student's *t*-test and *p* < 0.05 was considered significant. One-way Anova; Dunnett's Multiple Comparison Test was used to evaluate the impact of PI3K inhibitors in cytosolic vacuolization versus vehicle-treated controls. In figures, “*” stands for *p* < 0.05, “**” for *p* < 0.01, and “***” for *p* < 0.001.

## SUPPLEMENTARY MATERIAL FIGURES AND VIDEO




