# Supplementary material for: RAB7 counteracts PI3K-driven macropinocytosis activated at early stages of melanoma development
Source: Oncotarget. 2015 May 9;6(14):11848–62. doi: 10.18632/oncotarget.4055 (PMC4494909; doi:10.18632/oncotarget.4055)
Supplement: Supplementary file 1 [file oncotarget-06-11848-s001.pdf]

RAB7 counteracts PI3K-driven macropinocytosis activated at early stages of melanoma development

Supplementary Material

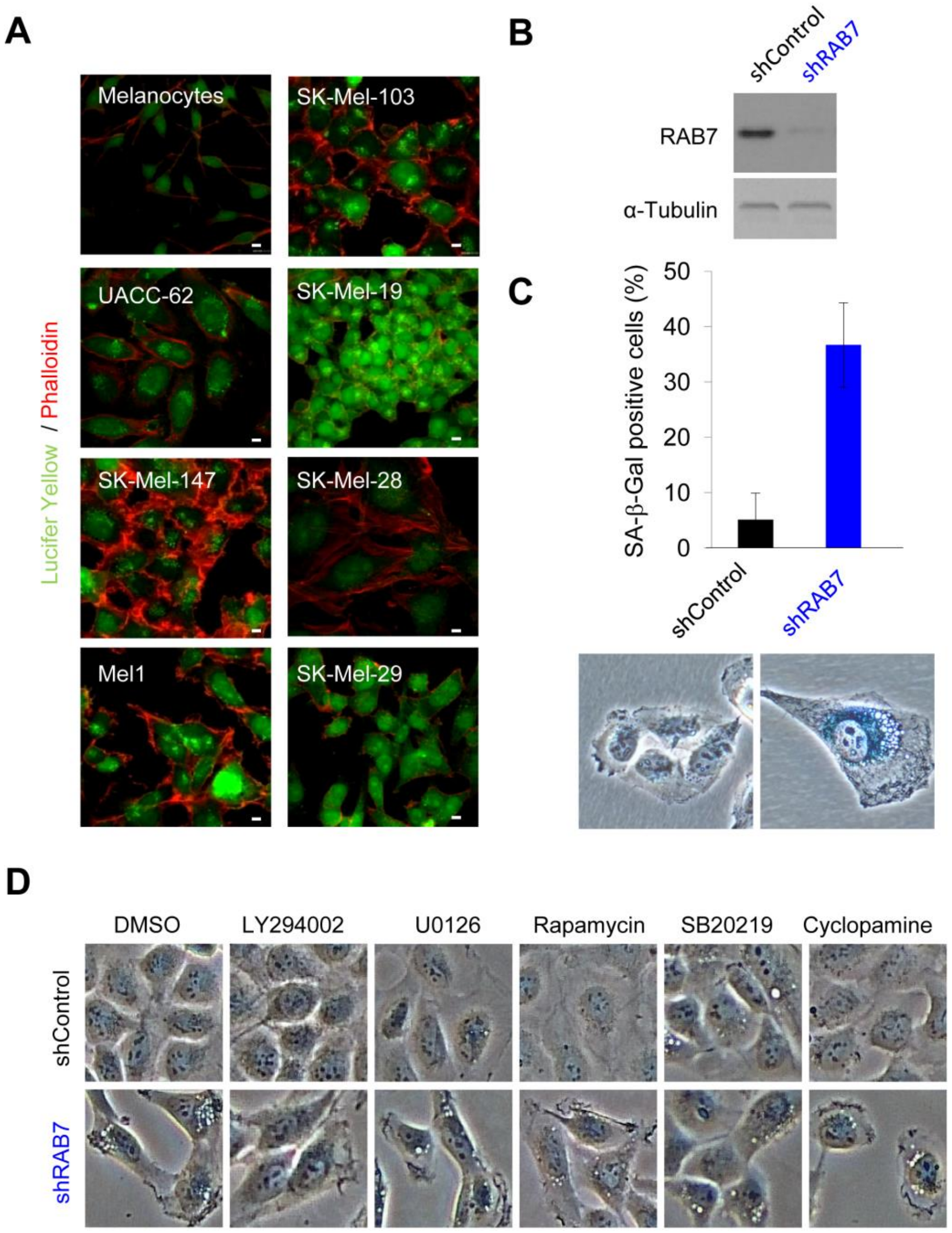

Figure S1: Vesicular trafficking in melanocytic cells. (A) Confocal visualization of constitutive fluid phase endocytosis in normal melanocytes and in the indicated melanoma cell lines. Shown is the uptake of the extracellular fluid marker Lucifer Yellow (green). Phalloidin staining (red) outlines cytoskeletal actin. (B) Immunoblots of total cell extracts isolated from SK-Mel-103 melanoma cell lines stably expressing control shRNAs (shControl) or RAB7 shRNAs (shRAB7), and probed for RAB7 or α-Tubulin (loading control). (C) Representative bright field micrographs showing SA-β-Gal stainings of shControl or shRAB7 SK-Mel-103 melanoma

cells. Bottom panels show the relative number of cells (means ± SEM) staining positive for the same cell populations. (D) Impact 10µM LY294002, 5µM U0126, 25nM Rapamycin, 5µM SB20219 or 10µM Cyclopamine on the cytosolic vacuolization induced by RAB7 downregulation in SK-Mel-103 melanoma cells. Shown are representative bright field images after 24h treatment of cells expressing controls shRNAs (shControl) or RAB7 shRNAs (shRAB7). Scale bars, 10µm.

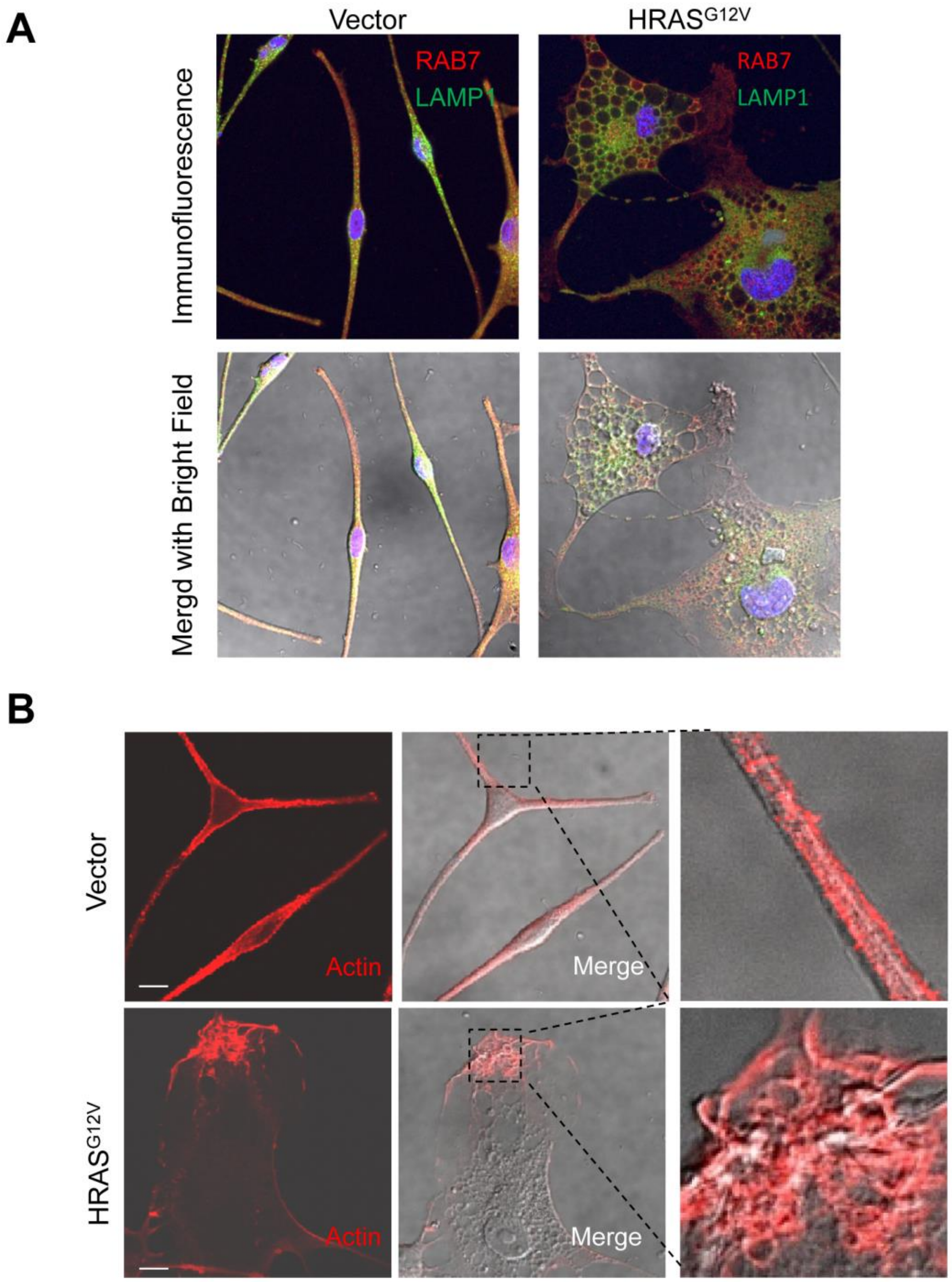

Figure S2: Impact of oncogenic RAS expression on the endolysosomal pathway. (A) Double immunofluoresce (IF) staining of the endolysosomal markers RAB7 (red) and LAMP1 (green) in primary human melanocytes transduced with empty $HRAS^{G12V}$-encoding vectors. Nuclei are counterstained with DAPI. Fluorescence and bright field-merged images are also included to visualize RAB7 decorating the large vacuoles induced by oncogenic HRAS. (B) Representative confocal micrographs of actin visualized by immunofluorescent imaging of phalloidin (red) in empty vector- or $HRAS^{G12V}$-expressing melanocytes. Middle vertical panels correspond to fluorescence photographs merged with DIC to visualize cellular surface. High magnification insets are included to visualize actin-rich ruffles in control vs $HRAS^{G12V}$-expressing cells. Scale bars, 10μm.

A

B

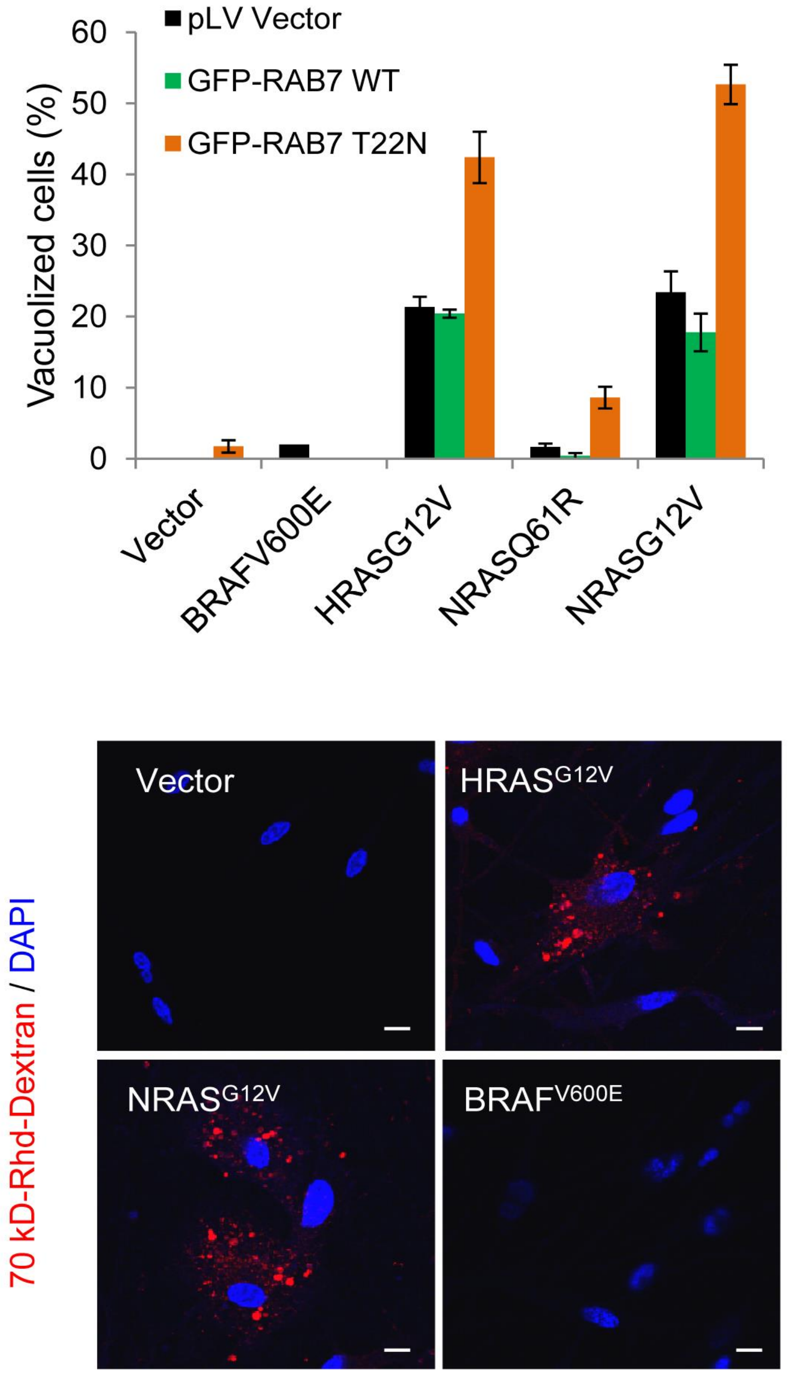

Figure S3: Macropinocytosis in control vs oncogenically-transduced melanocytes. (A) Quantification of vacuolized melanocytes expressing the indicated oncogenes, as well as wild-type (WT) or dominant negative (T22N) GFP-RAB7, or the corresponding empty vector control. (B) Representative confocal micrographs to visualize the uptake of 70kDa Rhodamine- Dextran (red) (2mg/mL, 2.5h) by the indicated melanocyte populations (i.e transduced with lentiviruses coding for empty vector or oncogenic $HRAS^{G12V}$, $NRAS^{Q61R}$ or $BRAF^{V600E}$). Nuclei are couterstained with DAPI. Scale bars, 10μm.

Supplementary Video 1: Dynamic changes in cell morphology in RAB7- depleted melanoma cells. Time lapse imaging of SK-Mel-103 cells stably expressing dominant-negative RAB7 (T22N). Images were captured at 10 min intervals in a Delta Vision RT microscope coupled to a CO_2 and temperature- controlled incubation chamber. Note the prominent and dynamic cytosolic vacuolization induced in the absence of functional RAB7.
